# Editorial: Towards an enactive approach to health: an integrative perspective which considers interdependence, agency, autonomy and participatory sensemaking in therapeutic phenomena

**DOI:** 10.3389/fpsyg.2024.1440751

**Published:** 2024-07-16

**Authors:** Mariana Lozada, Enara Garcia, Alejandro Chaoul, Paola D'Adamo

**Affiliations:** ^1^INIBIOMA, National Scientific and Technical Research Council (CONICET), National University of Comahue, Bariloche, Argentina; ^2^Department of Philosophy, University of Granada, Granada, Spain; ^3^University of Texas Health Science Center at Houston, Houston, TX, United States; ^4^ECyC IPEHCS, National Scientific and Technical Research Council (CONICET), National University of Comahue, Bariloche, Argentina

**Keywords:** enaction, health, affectivity, empathy, medicine

Health is frequently addressed from a dualistic perspective that separates mind and body. The biomedical paradigm tends to focus on observable and measurable symptoms, focusing on objective indicators of disease and relegating subjective experience to a mere epiphenomenon. Moreover, the medical system tends to assume that experts must solve health issues while patients passively receive prescriptions, leading to an epistemic asymmetry between the third-person perspective and patients first-person experience. The agency of the patient as the expert of their own health experience is often dismissed.

Over the last 30 years, the enactive approach has developed an integrative theory of cognition that overcomes limitations of neurocentric, cognitivists and reductionist viewpoints (Varela, 2000). Taking embodiment, environment and interpersonal relations in due account, the enactive approach defines sense-making as the process of meaning-making of living organisms with their environment, who evaluate, gauge, interpret, and act according to their self-maintenance, goals, and needs. Sense-making is deeply embodied in living beings' organization, which are intricately intertwined and interdependent with their environment. Cognition is thus embedded in a meaningful socio-ecological context in which interactions enable, scaffold or modulate meaning making in participatory sense-making processes.

Drawing on insights from organizational accounts in biology, dynamical systems theory and phenomenology, the enactive approach provides an integrative framework to address traditional issues in health studies, such as the demarcation between health and pathology, the multidimensionality of health, and empathy during the therapeutic process (Stilwell and Harman, 2019; De Haan, 2020; Arandia and Di Paolo, 2021). This approach emphasizes on the significance of agency and autonomy, contributing fruitful insights into the complex phenomenon of health. While medical systems tend to ignore the bidirectional interplay between local and global dynamics, the enactive perspective considers health as an emergent process, not reducible to local rules. Comprehension of how global and local levels interact could shed new light on the health problem. The enactive viewpoint helps us go beyond dualistic health approaches, raising awareness of the complex interrelations between organic, sensorimotor and intersubjective factors which underlie human experience (Toro et al., 2020; García and Arandia, 2022; Gauld et al., 2022; Cormack et al., 2023).

This Research Topic gathers seven research articles that addresses the health problem from an enactive perspective. It encompasses theoretical reflections, clinical trials and case studies in diverse health related fields. The proposals can be gathered into three guiding themes that traverse the enactive approach to health: (1) the relevance of subjectivity in assessment of health, (2) the understanding of the clinical treatment as participatory sense-making process (3) the relevance of developmental understanding of health to design prevention strategies. All these drive to an understanding of health as an emergent multidimensional process.

González-Grandón et al. propose an enactive, phenomenological, and ecological conceptualization on interoceptive processes, emphasizing the importance of understanding affectivity in the context of the living embodied self in dynamic interaction with the environment. Likewise, Sørvoll et al. propose that the enactive approach can complement theoretical frameworks of motor control and skill acquisition in pediatric physiotherapy by recognizing the child as a socially participating subject, emphasizing interaction processes and touch as a form of communication. The above-mentioned framework is put into practice in the study by Boge-Olsnes et al., which explores the experiences of women with chronic pelvic pain, demonstrating the importance of viewing the body as expressive and communicative while strengthening the therapeutic alliance. In the same vein, Hurissa et al. assess the effect of empathy training on healthcare providers, showing an increase in empathy levels, potentially promoting patients' autonomy and agency.

Lozada and D'Adamo share their experience about enactive interventions in children, which favored emotional regulation processes, their sense of agency, self-awareness and awareness of others, while helping to reduce chronic stress levels. Likewise, Mikhaylova et al. develop a psychometric scale to measure self-care autonomy and healthy habits in children, which advocates for prevention and adoption of good habits. Lastly, Weichold and Candiotto provide insights into the ethics of sense-making that have strong implications for individuals' wellbeing and their relationships with others. Sense-making, as an affective, and normative process, involves ethical considerations in issues concerning the demarcation between health and pathology, and in framing modes of interacting in therapeutic interventions. In conclusion, the current studies provide renewed evidence on how the enactive paradigm can contribute to further deepen and comprehend the complex phenomenon of human health.

## Author contributions

ML: Writing – original draft, Writing – review & editing. EG: Writing – original draft, Writing – review & editing. AC: Writing – original draft, Writing – review & editing. PD'A: Writing – original draft, Writing – review & editing.
